# Integrated CuO/Pd Nanospike Hydrogen Sensor on Silicon Substrate

**DOI:** 10.3390/nano12091533

**Published:** 2022-05-02

**Authors:** Ru Lin, Qi Hu, Zuolian Liu, Shusheng Pan, Zhifeng Chen, Wei Zhang, Zhiyu Liu, Shaolin Zhang, Chengyun Zhang

**Affiliations:** 1 School of Physics and Materials Sciences, Guangzhou University, Guangzhou 510006, China; rulinguangda@163.com (R.L.); 2112019060@e.gzhu.edu.cn (Q.H.); zuolian@gzhu.edu.cn (Z.L.); sspan@gzhu.edu.cn (S.P.); chenzf@gzhu.edu.cn (Z.C.); wzhang@gzhu.edu.cn (W.Z.); liuzhiyu@gzhu.edu.cn (Z.L.); 2 School of Electronics and Communication Engineering, Guangzhou University, Guangzhou 510006, China; 3 Research Center for Advanced Information Materials (CAIM), Huangpu Research Graduate School, Guangzhou University, Guangzhou 510555, China

**Keywords:** femtosecond laser, hydrogen sensor, CuO, Pd, Si nanospike, green process

## Abstract

A large area of randomly distributed nanospike as nanostructured template was induced by femtosecond (fs) laser on a silicon substrate in water. Copper oxide (CuO) and palladium (Pd) heterostructured nanofilm were coated on the nanospikes by magnetron sputtering technology and vacuum thermal evaporation coating technology respectively for the construction of a p-type hydrogen sensor. Compared with the conventional gas sensor based on CuO working at high temperature, nanostructured CuO/Pd heterostructure exhibited promising detection capability to hydrogen at room temperature. The detection sensitivity to 1% H_2_ was 10.8%, the response time was 198 s, and the detection limit was as low as 40 ppm, presenting an important application prospect in the clean energy field. The excellent reusability and selectivity of the CuO/Pd heterostructure sensor toward H_2_ at room temperature were also demonstrated by a series of cyclic response characteristics. It is believed that our room-temperature hydrogen sensor fabricated with a waste-free green process, directly on silicon substrate, would greatly promote the future fabrication of a circuit-chip integrating hydrogen sensor.

## 1. Introduction

As an important industrial chemical and green energy, hydrogen provides energy support for global sustainable development. The production, transportation and storage of hydrogen energy involve various fields, such as automobile, fuel cell, rocket engine, chemical industry, aircraft, semiconductor manufacturing and metallurgy [1], which has attracted extensive attention [2]. In addition, hydrogen can also be used effectively for a variety of disease treatment and may play an important role in medical and biological research in the future [3]. However, hydrogen is invisible, tasteless, and highly flammable and explosive when the concentration of hydrogen in air is higher than 4%. Therefore, it is necessary to manufacture sensors that can detect hydrogen leakage [4,5]. At present, there are many types of hydrogen sensors based on electrochemistry [6], optics [7,8], surface acoustic wave [9], catalysis [10,11], mechanics [12] nano resistance [13,14,15] and so on. The semiconducting metal oxide sensor has become a promising candidate for hydrogen detection due to its advantages of high sensitivity, low cost, abundance, chemical stability, easy fabrication, and environmental protection [1]. For example, oxides of various metals such as tungsten, titanium, zinc [4,16,17,18,19,20,21,22] are used in the constructions of hydrogen sensors. Especially the hydrogen sensors based on CuO, a non-toxic and low-cost p-type semiconducting metal oxide with a band gap of 1.2~1.9 eV, have attracted much attention [23,24,25]. CuO can be used not only for hydrogen sensing, but also as a promising candidate for the detection of low-concentration volatile organic compounds. CuO nanostructures with large surface-to-volume ratios have been used to detect various toxic gases such as carbon monoxide, hydrogen sulfide and amines at different operating temperatures [26].

It is worth noting that the application of various nanostructures of semiconductor materials can improve the performance of hydrogen sensors due to their high surface area–volume ratio [27]. For example, it has been studied to enhance the sensing response by constructing CuO nanostructures, such as nanorods [28], nanoflakes [29], nanofilms [30], nanowires [31] and nano-bitter gourd [32]. Meanwhile, many studies have demonstrated the improved sensitivity and selectivity of the hydrogen sensor by incorporating other noble metal nanostructures as catalysts, such as Au [33], Ag [34], Pd [35] and Pt [36]. It is reported that the deposition of platinum (Pt) and Pd nanoparticles (NPs) on p-type silicon nanowalls (Si NWs) could prominently enhance its hydrogen sensor performance [37]. For the hydrogen sensors based on material nanostructures, the catalysis of noble metals, especially Pd, plays an excellent role in improving their hydrogen sensing performance. Pd NPs were also decorated on the silicon nano-horns by UV reduction method for hydrogen sensing [38]. Pd-based hydrogen sensors have a unique advantage because Pd nanostructures can act as a catalytic to break the H-H bond in diatomic hydrogen, making the monatomic hydrogen diffuse into the material [39]. Moreover, various morphologies of Pd can dissolve more than 600 times its own volume of hydrogen, but can hardly dissolve other common gases such as oxygen, nitrogen, nitric oxide and carbon oxide, which makes Pd the most selective hydrogen sensing material [39]. Therefore, some researchers improved the sensitivity of hydrogen sensor by modifying CuO nanorods with Pd/Pt [40], magnetron sputtering Pd coated CuO film [22] or Pd nanoparticles functionalization on ZnO nanowires [41], etc. It is an effective way to incorporate the semiconductor and Pd nanostructures to optimize the performance of hydrogen sensors.

As we know, there are many ways to prepare nanostructured hydrogen sensors. For example, Gao et al. partially reduced the hollow shell of PdO by chemical solution to form catalyst Pd NPs, and manufactured a high-performance hydrogen sensor with long-term stability [42]. Nanoparticles of different materials and morphologies have also been used for the manufacture of high-performance hydrogen sensors by using various techniques, including microwave-assisted synthesis [43], laser deposition technique [44], electrospinning and UV radiation [45], rapid thermal evaporation [46] or pulsed laser ablation (PLA) [47]. Moreover, a combination of two different methods for the preparation of nanostructures, such as metal assisted chemical etching and pulsed laser deposition (PLD) [48] or traditional photolithography and the wet stripping process [49], is also widely used in the field of hydrogen sensor. In addition, there are many nanofilms prepared by magnetron sputtering for the research of hydrogen sensors [10,40,50,51]. However, above-mentioned methods involved with either chemical treatment or complicated process presenting low compatibility with current silicon technology. Among the various fabrication methods of nanostructures, fs laser can directly induce unique nanostructures on the surface of almost all bulk materials including Si and SiO_2_ substrates due to its ultrashort pulse, high peak power and breaking diffraction limit [52,53]. Therefore, fs laser micromachining technique is an effective, competitive method for preparing nanostructures to construct hydrogen sensor.

Generally speaking, gas sensors based on metal oxides need high operating temperature to obtain excellent sensing performance. However, the high operating temperature will lead to high power consumption, system incompatibility and performance degradation [31]. Most hydrogen sensors based on CuO nanostructures need to work at high temperatures ranging from 200 °C to 400 °C [22,24,28,32,40,54]. Therefore, the sensing performance of hydrogen sensors working at room temperature needs to be further explored. In this study, fs laser is used to induce dense and randomly distributed nanospike arrays on the surface of silicon substrate in water, which acts as a nanostructured template. Then, CuO and Pd nanofilms were deposited on the surface of nanospike arrays by magnetron sputtering and vacuum thermal evaporation coating technology, respectively. It was found that the sensor has good response and reusability to hydrogen at room temperature. The application of Si substrate is compatible with the current semiconductor fabrication technology, which lays a good foundation for the future preparation of a hydrogen-sensor integrated electronic chip. The development of silicon-based nanostructured hydrogen sensor chip can undoubtedly improve the stability and reliability of the device and reduce the power consumption of the device.

## 2. Materials and Methods

### 2.1. Fabrication of Heterostructured Nanofilm

A silicon wafer with size of 16 mm × 16 mm × 0.5 mm was ultrasonically cleaned with deionized water for 5 min and then dried with nitrogen before laser treatment. The sample was placed in distilled water at a depth of 2 mm and fixed on a three-dimension (3D) electric displacement platform. The 3D displacement platform was controlled by computer to move accurately in XYZ directions. A fs amplifier (Legend Elite HE, Coherent, CA, USA) output a horizontal polarization pulse of 100 fs at 1 kHz repetition frequency with central wavelength of 800 nm. The laser beam with a Gaussian profile was focused by using a lens with focusing length of 150 mm. The laser power was 4.5 mW. The linear scanning speed was 1 mm/s and the scanning interval was 22 μm.

The laser treated silicon wafer was ultrasonically cleaned with deionized water for 1 min and then dried with nitrogen again. A layer of CuO nanofilm was deposited by high vacuum magnetron sputtering (JCP-350, Beijing Technol Science, Beijing, China). The magnetron sputtering time was set to 30 min and the volume ratio of Ar:O_2_ was 32:4. Afterwards, another layer of Pd nanofilm was deposited by using vacuum thermal evaporation coating technology. An amount of 8.6 mg Pd particles with purity of 99.99% was placed on the evaporation boat of the vacuum thermal evaporation coater (ZHD-300, Beijing Technol Science, China) for the coating. The evaporation current was 130 A and the evaporation time was 2 min.

### 2.2. Morphological and Structural Characterization

The surface morphologies and elemental distribution of Si nanospike before and after coating were observed by using a field emission scanning electron microscope (SEM) (ZEISS Gemini 500, Carl Zeiss, Baden-Württemberg, Germany) equipped with an energy-dispersive X-ray spectrometer (EDS) (X-Max, Oxford, UK). The operating voltage was 2 kV. The X-ray diffraction (XRD) analysis was carried out by using X-ray diffractometer (X’PERT PRO, PAnalytical, Netherlands) equipped with a Cu-Kα radiation source (λ = 1.5405 Å). 

### 2.3. Hydrogen Response Measurement

**Figure 1:**
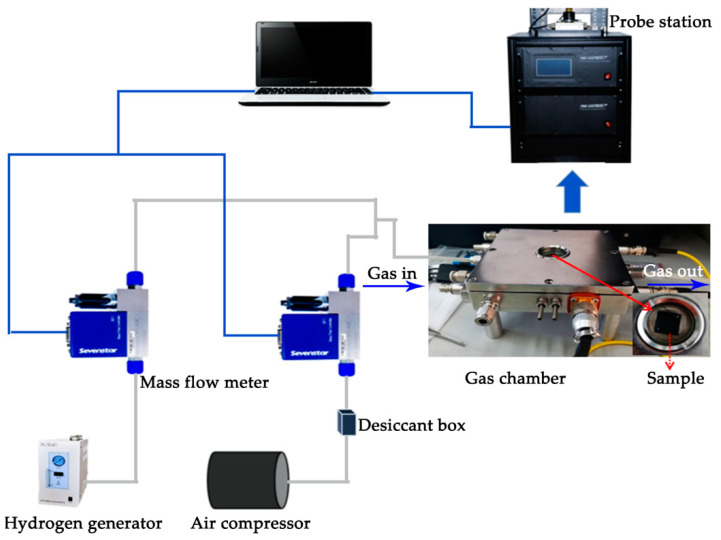
Schematic diagram of the hydrogen sensor measurement system.

The schematic diagram of hydrogen sensor measurement system is shown in Figure 1. A hydrogen generator (SPH-500, BCHP Analytical Technology Institute, Beijing, China) was used to provide hydrogen with purity of 99.999% and pressure of 0.3 MPa. An air compressor and desiccant box were used to obtain dry air as dilution gas. The test system controlled the flow rate of air and hydrogen through two mass flow controllers (MFC, D07-7B, Qixing Huachuang, Beijing, China) to configure a certain concentration of hydrogen. The configured target gas then flowed through the pipeline into the test chamber (100 mL). The fabricated sensor using the indium as electrodes was placed in the test chamber and electrically contacted by two tungsten probes. The current–voltage (I/V) characteristics of the sensor at different hydrogen concentrations were recorded by the photoelectrical comprehensive test platform (CGS-MT, Sino Aggtech, Beijing, China). The measurement voltage of the test system was set to 1 V, and the data were collected and stored in the computer. The sensitivity of the sensor is defined as $S=\left(R_{g}-R_{a}\right)/R_{a}\times 100\%$, where $R_{a}$ is the resistance of the sensor in air, and $R_{g}$ is the resistance of the sensor in different concentrations of hydrogen.

## 3. Results and Discussion

### 3.1. Characterization of Laser Treated Si Surface

**Figure 2:**
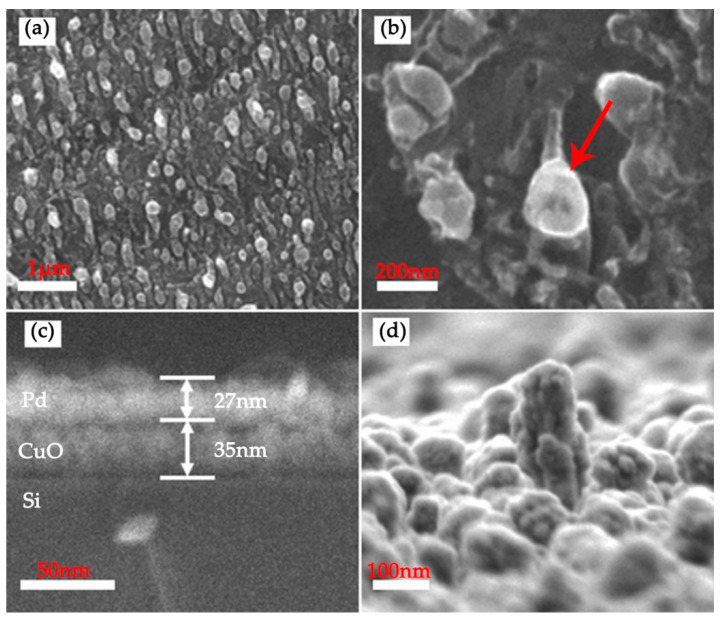
(**a**,**b**) SEM images (top view) of the Si nanospike before coating CuO and Pd heterostructured nanofilms; (**c**,**d**) SEM images (cross-sectional view) of the Si nanospike after coating CuO and Pd heterostructured nanofilms.

**Figure 3:**
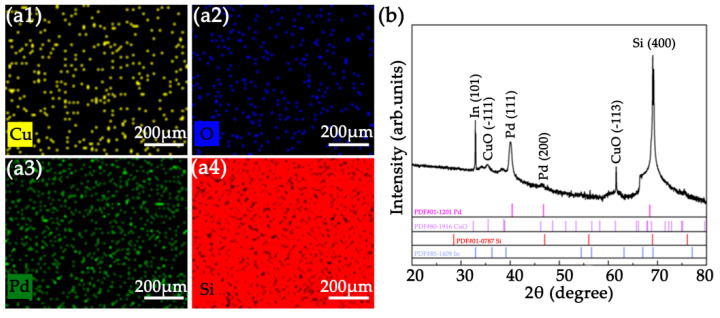
(**a**) EDS mapping images of the Si nanospike after coating heterostructured CuO and Pd nanofilms. (**a1**) Cu element, (**a2**) O element, (**a3**) Pd element, (**a4**) Si element; (**b**) XRD pattern of the heterostructured CuO/Pd nanofilm on Si nanospike.

As shown in Figure 2, the surface morphology of the laser treated sample was characterized by SEM before and after coating heterostructured CuO/Pd nanofilms. It can be clearly observed that a large-area and randomly distributed nanospike structure was directly induced on the surface of the silicon substrate, which acts as nanotemplate and greatly improves the surface area of the hydrogen sensor, enabling the sensor to detect low concentration of hydrogen, as shown in Figure 2a. The magnified SEM image was shown in Figure 2b. The diameter of the nanospike indicated by red arrow was about 284 nm. An obvious two-layer structure was observed from the SEM image of cross-section, as shown in Figure 2c, in which the thicknesses of CuO and Pd nanofilms were about 35 nm and 27 nm, respectively. Moreover, Figure 2d shows that CuO and Pd nanoparticle clusters were formed on the surface of Si nanospike structures. Laser induced nanospikes on silicon wafer is expected to serve as a nanotemplate to grow nanostructured CuO and Pd with large surface–volume ratio. Furtherly, Figure 3a displays the elemental distribution of the Si nanospike after coating heterostructured CuO and Pd. The elemental mapping indicated that all the elements, such as Pd, Cu, Si and O, were present and no other impurity was observed. Furthermore, the structural information of the coating was characterized by X-ray diffraction analysis. As shown in Figure 3b, the X-ray diffraction pattern indicated that the heterostructured nanofilm was composed of CuO (PDF#80-1916) and Pd (PDF#01-1201), which is in good agreement with the result of SEM observation. The peaks of In (101) and Si (400) stemmed from the electrode material and monocrystalline substrate, respectively. Thus, it is evident that the formation of CuO and Pd heterostructure using fs treated Si as nanotemplate is successfully achieved. It is worthwhile to note that the formation of Si nanospike as well as the followed deposition of CuO and Pd are green, free of chemical process, presenting full compatibility with current silicon technology.

### 3.2. Hydrogen Sensor Performance

**Figure 4:**
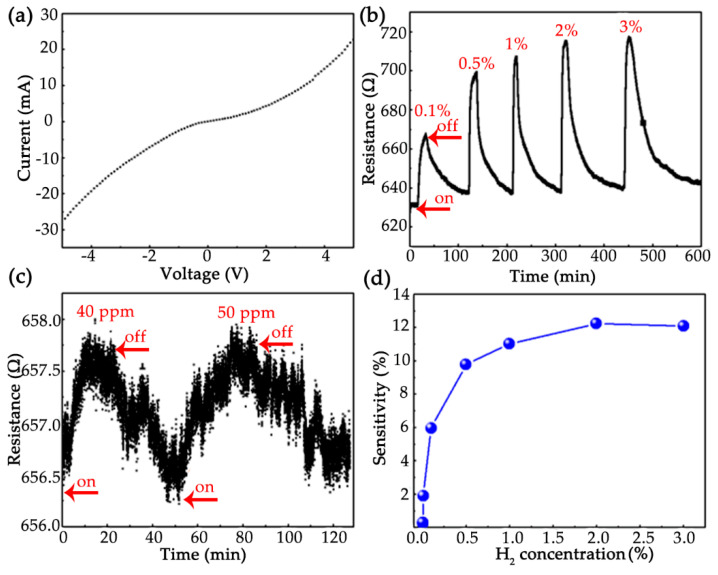
(**a**) I/V characteristic curve of the sensor in air; (**b**) Sensing response at different hydrogen concentrations (0.1–3%); (**c**) Detection limit of hydrogen concentration; (**d**) Sensitivity of sensor at different hydrogen concentrations. The on and off symbols in the figures indicated that the hydrogen flow was turned on and off respectively.

The hydrogen sensor measurement system is shown in Figure 1. The I/V characteristic curve of the sensor in air is shown in Figure 4a which indicates an ohmic contact. In order to test the performance of the hydrogen sensor, we measured the sensing response in the dynamic range of 0.1–3% hydrogen concentration at room temperature (Figure 4b). Firstly, the dry air flowed through the gas chamber and the intrinsic resistance of the sensor was automatically recorded. Thereafter, the configured target gas with different concentration precisely controlled by hydrogen mass flowmeters was introduced into gas chamber. Upon exposure to the hydrogen, it was observed that the resistance of the sensor quickly increased, and tended to stabilize after reaching a maximum. Once the hydrogen flow was turned off, the resistance of the sensor decreased gradually and returned to the intrinsic resistance, as shown in Figure 4b. The detection limit of the sensor for lower hydrogen concentration was obtained by reducing the flow rate of hydrogen. The detection limit at room temperature was 40 ppm, as shown in Figure 4c, indicating that the sensor can detect very low concentration of hydrogen. Figure 4d and Table 1 summarize the sensitivity, response time and recovery time of the sensor as a function of the increasing hydrogen concentration. Our sensor presented a broad detection range of hydrogen concentrations from tens of ppm to several percentage. The saturation occurred when the concentration increased over 2% while the sensitivity was 12.2%.

**Figure 5:**
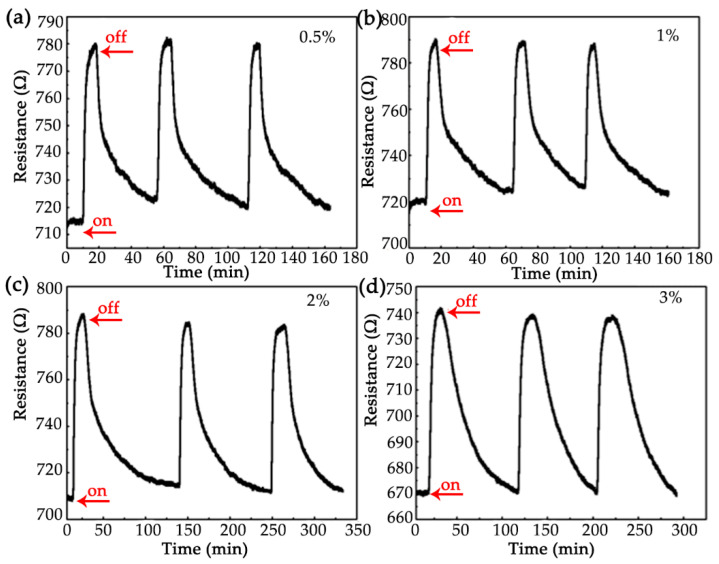
Cyclic response of the sensor at different hydrogen concentrations (**a**) 0.5%; (**b**) 1%; (**c**) 2% and (**d**) 3%.

**Figure 6:**
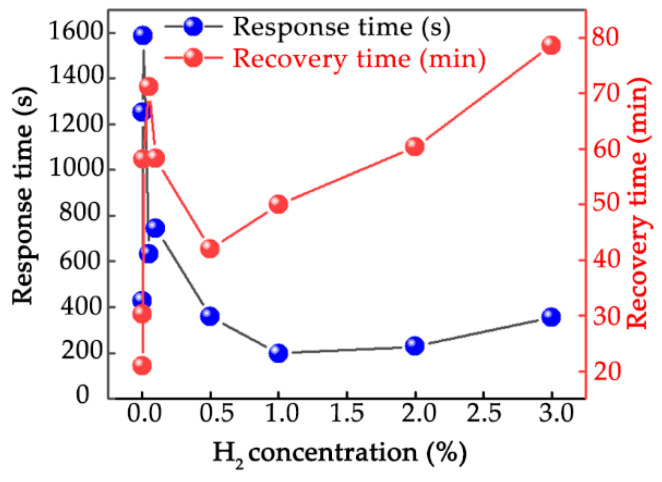
Response and recovery time of sensor at different hydrogen concentrations.

In order to characterize the repetitive property of the sensor, the cyclic responses of the sensor were tested toward the hydrogen with concentration of 0.5% (Figure 5a), 1% (Figure 5b), 2% (Figure 5c) and 3% (Figure 5d), respectively. It can be found that the sensor has good cyclic response at room temperature, which indicates that the sensor has high reusability. The response and recovery time of the sensor at different hydrogen concentrations are shown in Figure 6. It can be found that when the hydrogen concentration is 1%, the response time is 198 s. Although the recovery time is relatively long, it is of great significance to study the working condition at room temperature compared with the hydrogen sensor based on p-type semiconducting metal oxide working at high temperatures.

We also compared the performance parameters of our sensor with those of sensors in the literature, as shown in Table 2, Compared with other CuO based hydrogen sensors working at high temperature, our sensor has good hydrogen sensitivity at room temperature, which shows that depositing CuO and Pd on fs laser-treated Si substrate is an excellent choice for hydrogen detection. Although the performance of our hydrogen sensor is slightly lower than that of other nanomaterial-based hydrogen sensors working at room temperature, our preparation method is convenient, fast, pollution-free and fully compatible with current silicon technology. The power consumption of the sensor is very low. For example, when detecting hydrogen at a concentration of 1%, the power consumption varies from 1.26 mW to 1.39 mW.

Selectivity and long-term stability are critical important to the sensor performance. The heterostructured CuO/Pd nanofilm sensor was tested to benzene, ethanol, acetone, methanol and ammonia, respectively. The results revealed that our sensor presented no observable response toward these gases at room temperature owing to the deficiency of the activation energy. The long-term stability test was also carried out. After 60 days of exposure to air, the sensor’s detection sensitivity to 2% hydrogen still reached 11.2%, indicating that our sensor has good long-term stability.

### 3.3. Sensing Mechanism

In general, the strategies of utilizing p-type oxide semiconductor for practical gas sensors application include: (1) the preparation of nanostructures with different morphologies, (2) doping and decorating noble metals or metal oxide catalysts in the oxide semiconductors [27] or (3) constructing a heterojunction with n-type semiconductor. These methods can improve the gas response of p-type oxide semiconductor gas sensors. 

Herein, the sensing response mechanism is contributed to the change of the electrical resistance of the hydrogen sensor upon hydrogen exposure. When the sensor is exposed to air, the oxygen molecules in air are adsorbed on the CuO surface and ionized into reactive oxygen species $O_{2}^{-}$, $O^{-}$ or $O^{2-}$. At room temperature, it mainly exists in the form of $O_{2}^{-}$ [37,57], as shown in Equations (1) and (2).

(1) $$O_{2}\left(\mathrm{gas}\right)\to O_{2}\left(\mathrm{ads}\right)$$

(2) $$O_{2}\left(\mathrm{gas}\right)+e^{-}\to O_{2}^{-}\left(\mathrm{ads}\right)$$

**Figure 7:**
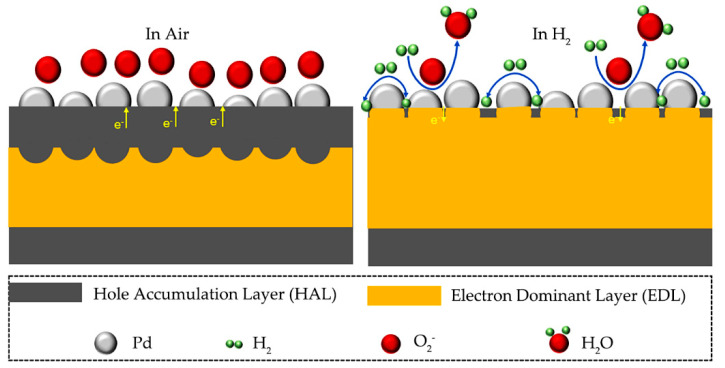
Schematic diagram of response mechanism.

The response mechanism of hydrogen sensor is shown in Figure 7. When Pd and CuO are in close contact in air, electrons flow from CuO to Pd, which leads to the expansion of the hole accumulation layer (HAL) and the contraction of the electron depletion layer (EDL) at the interface between Pd and CuO. When CuO was exposed to hydrogen, H_2_ spillover effect occurred due to the decorated catalytic noble metal. In this case, catalytic metal provides large number of active sites for the adsorption of H_2_ molecules. Due to the high solubility and diffusivity of H_2_ molecules, the adsorbed H_2_ molecules are dissociated into atomic species, and then rapidly diffuse through catalytic Pd. The adsorbed hydrogen reacts with the adsorbed oxygen ions according to Equation (3), and the released electrons quickly combine with the holes in HAL, resulting in the decrease in the hole concentration and thinning of the HAL thickness. The holes are the majority carriers in p-type CuO semiconductor. Due to the annihilation of the holes, the resistance of CuO semiconductor increases, resulting in the response of gas sensor. When hydrogen is turned off and there is only air, most H_2_ molecules are desorbed from the Pd layer. Therefore, the previously injected electrons will leave the CuO film to restore the resistance of the sensor.

(3) $$2H_{2}+O_{2}^{-}\left(\mathrm{ads}\right)⟶2H_{2}O+e^{-}$$

Beside the receptor function and the synergistic effect of Pd and CuO, the fs laser induced nanostructure also played an important role in the hydrogen sensing behavior. As a comparison, we fabricated a CuO/Pd hydrogen sensor on smooth Si substrate without fs laser treatment. Under the same condition, we tested the cyclic response of the sensor at a hydrogen concentration of 1%. The results show that the repeatability shifts and the corresponding sensitivity is unstable. In addition, the hydrogen sensor has no response to hydrogen at a concentration of 0.1%, and its detection limit of hydrogen is much higher than that of CuO/Pd with nanospikes structure. It can be seen from Figure 2, the surface of the substrate is covered with randomly distributed nanospike structures, which greatly improves the surface area of the sensor. The sensor has a high surface area to volume ratio, which can provide more active sites for hydrogen, greatly improving the hydrogen sensitive response of the sensor.

## 4. Conclusions

We used an fs laser ablation strategy to directly induce large area of randomly distributed nanospike structures on silicon substrate as a nanotemplate conveniently and quickly. A 35-nm-thickness CuO layer and another 27-nm-thickness Pd layer were decorated on the surface of nanospike by magnetron sputtering and vacuum thermal evaporation coating technology, respectively. The substrate surface covered with nanospikes greatly improves the surface area of the sensor, which is helpful to improve the hydrogen sensing response of the sensor. The sensitivity of the sensor to 1% hydrogen is 10.8%, the response time is 198 s at room temperature and the hydrogen concentration can be detected as low as 40 ppm. It was found that the sensor had good recycling performance. There are few research studies on hydrogen sensors based on p-type semiconductor oxides and almost all of them work at high temperature. Therefore, it is of great significance to study hydrogen sensors based on p-type semiconducting metal oxides working at room temperature. Compared with other methods for preparing nanostructures, the fs laser direct writing technology is more convenient and rapid. It is worthwhile to note that the fs laser micromachining technique directly forming nanostructures on substrate is free of chemical use and totally compatible with current Si process. Moreover, the use of Si substrate lays a good foundation for the preparation of hydrogen-sensor integrated electronic chips in the future. The development of silicon-based nanostructured hydrogen sensor chip can undoubtedly improve the stability and reliability of the device, and reduce the power consumption of the device.

## Figures and Tables

**Table 1:** The sensor response time (t_res_), recovery time (t_rec_) and sensitivity (S) of the sensor toward different concentrations of hydrogen (H_2_ conc.).

H_2_ conc.	t_res_ (s)	t_rec_ (min)	S (%)
40 ppm	428	30.15	0.23
50 ppm	1250	20.83	0.28
0.01%	1585	58.01	1.90
0.05%	632	71.08	4.26
0.1%	743	58.26	5.95
0.5%	358	41.98	9.76
1%	198	49.86	10.80
2%	229	60.28	12.20
3%	355	78.56	12.07

**Table 2:** Comparison of performance parameters with reported data.

Material	H_2_ conc.	t_res_	t_rec_	S *	T. (°C)	Ref.
CuO NW networks	100 ppm	60 s	2 s	340	300	[24]
Nb_2_O_5_ Nps/CuO Nanorod	0.50%	161 s	163 s	217.05%	300	[28]
Pd/CuO Nanorod	1000 ppm	10 min	16 min	4.5	200	[40]
Nano-bitter gourd CuO	100 ppm	150 s	1016 s	175%	200	[32]
Pd/SnSe/SiO_2_/Si	0.10%	73.1 s	23.7 s	3225	RT	[55]
TiO_2_ Nanofibers	1000 ppm	1.49 min	0.52 min	63	RT	[56]
Pd/CuO/Si	1%	198 s	49.86 min	10.80%	RT	This work

* The sensitivity is calculated in different ways.

## Data Availability

The data presented in this study are available on request from the corresponding author.
